# Gender, Age, Geographical Area, Food Neophobia and Their Relationships with the Adherence to the Mediterranean Diet: New Insights from a Large Population Cross-Sectional Study

**DOI:** 10.3390/nu12061778

**Published:** 2020-06-15

**Authors:** Stefano Predieri, Fiorella Sinesio, Erminio Monteleone, Sara Spinelli, Marta Cianciabella, Giulia M. Daniele, Caterina Dinnella, Flavia Gasperi, Isabella Endrizzi, Luisa Torri, Tullia Gallina Toschi, Alessandra Bendini, Ella Pagliarini, Camilla Cattaneo, Rossella Di Monaco, Paola Vitaglione, Nicola Condelli, Monica Laureati

**Affiliations:** 1 Institute for Bioeconomy, CNR, National Research Council, Via Gobetti 101, 40129 Bologna, Italy; stefano.predieri@ibe.cnr.it (S.P.); giuliamaria.daniele@ibe.cnr.it (G.M.D.); 2 CREA, Council for Agricultural Research and Economics, Research Center Food & Nutrition, Via Ardeatina 546, 00178 Rome, Italy; fiorella.sinesio@crea.gov.it; 3 Department of Agriculture, Food, Environment and Forestry (DAGRI), University of Florence, Via Donizetti 6, 50144 Florence, Italy; erminio.monteleone@unifi.it (E.M.); sara.spinelli@unifi.it (S.S.); dinnella@unifi.it (C.D.); 4 Center Agriculture Food Environment University of Trento via E. Mach 1, 38010 San Michele all’Adige, Italy; flavia.gasperi@fmach.it; 5 Research and Innovation Centre, Fondazione Edmund Mach, Via E. Mach 1, 38010 San Michele all’Adige, Italy; isabella.endrizzi@fmach.it; 6 University of Gastronomic Sciences, Piazza Vittorio Emanuele 9, 12042 Pollenzo, CN, Italy; l.torri@unisg.it; 7 Department of Agricultural and Food Sciences, Alma Mater Studiorum—University of Bologna, piazza Goidanich 60, 47521 Cesena (FC), Italy; tullia.gallinatoschi@unibo.it (T.G.T.); alessandra.bendini@unibo.it (A.B.); 8 Department of Food, Environmental and Nutritional Sciences, University of Milan, Via Celoria 2, 20133 Milano, Italy; ella.pagliarini@unimi.it (E.P.); camilla.cattaneo@unimi.it (C.C.); monica.laureati@unimi.it (M.L.); 9 Department of Agricultural sciences, University of Naples Federico II, via Università 100, 80055 Portici (NA), Italy; dimonaco@unina.it (R.D.M.); paola.vitaglione@unina.it (P.V.); 10 Department of Biology, DBAF, University of Basilicata, Via Nazario Sauro 85, 85100 Potenza, Italy; condelli@hotmail.it

**Keywords:** diet quality, food rejection, healthy eating, Mediterranean eating pattern, socio-demographics

## Abstract

The Mediterranean diet (MD) is associated with many health benefits. The association between the MD and food neophobia (FN) is still unexplored in adults. The present cross-sectional study was aimed to explore the relationships between adherence to the MD, FN, and sociodemographic variables in a large Italian cohort. Familiarity and frequency use (FFI) of prototypical and non-prototypical Mediterranean foods were used to calculate a new adherence index: the Italian Taste Mediterranean Index (ITMI). The FFI of all Mediterranean foods increased with age, while butter, soft drinks, red/cured meat, and sweets were more common in younger people. Accordingly, ITMI increased with age (F_2,2384_ = 54.11; *p* < 0.0001). Women recorded a higher ITMI (6.70) than men (6.10). Individuals with high FN showed higher FFI for soft drinks and sweets and lower ones for most typical MD foods, than individuals with low FNs. A decrease of ITMI was recorded with the increase of the FN(F_2,2384_ = 22.84; *p* < 0.0001). With ageing, ITMI increased even in individuals with a high FN. The results suggest that FN may negatively affect adherence to the MD, lowering its potential health benefits, in the adult population. Monitoring of food habits, dietary education, and anxiety management, may be valuable tools to control FN and support the adherence to the MD.

## 1. Introduction

Evidence shows that adherence to the Mediterranean diet (MD) is negatively associated with the risk of chronic diseases and all-cause mortality [1,2,3,4,5], improving a series of metabolic processes in the body [6,7]. Moreover, the MD is considered a sustainable diet [8] and was acknowledged as an Intangible Cultural Heritage of Humanity by UNESCO [9] in 2013.

The MD consists of a high consumption of fresh or dried fruit and vegetables, legumes, and whole grain cereals with moderate consumption of fish, dairy, and meat and a low-to-moderate amount of red wine during meals and the use of olive oil as the main source of fat [3].

The traditional MD, as described by Ancel Keys [10] in the “Seven Countries Study”, has changed over time, influenced by multiple social, economic, and behavioral changes in the populations. In an attempt to promote the adoption of the MD also outside the Mediterranean area [11], several indexes of adherence have been proposed [12]. However, in the last decades, concomitantly with an increased awareness of the MD’s health benefits, a decline in adherence, especially in Mediterranean populations, has occurred [13,14,15].

Addressing how consumers’ personal characteristics affect adherence to the MD might aid in understanding the causes of the discrepancy between consumers’ awareness of healthy eating and its practice as well as in designing strategies promoting healthy food choices.

One factor that negatively influences individual diet is food neophobia [16], i.e., the fear of trying new and unknown foods [17]. Individuals with high food neophobia are more selective with respect to food; therefore; they reduce dietary variety and are predisposed to inadequate nutrient intake [16]. For example, food neophobia is negatively associated to daily intake and liking of fruit and vegetables [18,19] as well as of food of animal origin, especially fish [20]. Recent evidence has shown that the effect of food neophobia may extend beyond rejection of unfamiliar/unusual foods to encompass many commonplace food items [21,22], reducing the nutritional quality of an overall diet and increasing metabolic disease risk [23] and obesity [24]. For instance, Sarin and co-workers [23] have recently demonstrated in a large population cohort of Finnish and Estonian adults that food neophobics have lower scores of a healthy Nordic diet and a higher incidence of coronary heart disease and type 2 diabetes, as assessed by health-related biomarkers.

Food neophobia is a heritable trait by up to 78% [25]. This behavioral trait is especially evident during childhood as a developmentally appropriate response against the ingestion of new and potentially toxic foods [23]; however, food neophobia has a high prevalence also among adults [21,22,26,27], and its association with individual factors, such as age, gender, personality features, living area, education level, and socioeconomic status, has been reported [28,29].

Although the effects of food neophobia on dietary aspects have been well documented, especially in children, to the best of our knowledge, the association between this behavioral trait and the adherence to the MD is still unexplored in adults. Recent studies have shown that personality traits impact differently on food preferences and choices with food neophobia being one of the major psychological barriers to healthy eating [30,31]. Two studies addressed this issue in school-aged children [32,33] and adolescents [32], evidencing a negative correlation between food neophobia and adherence to the MD. Whether this outcome applies also to the adult population is unknown, considering the fact that food preferences and consumption evolve with age as a result of increased exposure to a variety of foods and beverages [34]. Moreover, one of the limits of previous research on food neophobia is the reduced number of subjects involved. Large-scale studies are important to confirm or refute trends observed in smaller-sized research. Considering the implications of food neophobia in public health policies and the lack of information on the association between this personality trait and food consumption analyzed within a specific dietary pattern and in a given cultural framework, the main aim of the present cross-sectional study was to explore adherence to the MD, including diet diversification [35,36], in a large population of adults with different levels of food neophobia. Data from the familiarity and frequency use of pasta, fruits, vegetables, legumes, fish, red and cured meat, vegetable and animal fats, dairy products, alcohol, soft drinks and sweets were used to calculate a new index of adherence to the MD and verify its association with different levels of food neophobia also considering the relative contribution of demographic variables such as age, gender, and geographical area.

Since food neophobia is associated with a reduction in the consumption of fruit, vegetables, and fish, which are among the essential constituents of the MD, we hypothesized that food neophobia is negatively correlated to the adherence of that dietary pattern.

## 2. Material and Methods

The Italian Taste (IT) project is a large-scale study aimed at exploring the associations among biological, genetic, physiological, sociocultural, psychological, and personality-related factors, describing the dimensions of food liking, preference, behavior, and choice as well as their relevance in determining individual differences within a given food culture framework [37]. The IT project is a 3-year research project started in 2014 by the Italian Sensory Science Society (SISS). It engaged, on a voluntary basis, 58 SISS members working in 19 sensory laboratories of public and private organizations across the country and aimed at studying Italian consumers’ food liking, preference, and choice. This study involved online and in-sensory-lab testing sessions. The present cross-sectional study on the MD and food neophobia includes only a selection of IT project tests. For a complete description of the testing and further details on the definition of the IT project procedures, see Monteleone et al. [37].

### 2.1. Participants

Data were collected on 2449 Italian consumers balanced for age (age range: 18–60 years), gender, and residence in four geographical areas in Italy: North-East (NE), North-West (NW), Central (CE), South, and Islands (SO) (see details in Section 3.1). The database was revised to identify and delete missing values (*n* = 33). Statistics were performed on the remaining 2416 respondents.

Details on participants’ recruitment are reported in Monteleone et al. [37]. Briefly, participants were recruited by means of announcements published on social networks (i.e., Facebook), articles published in national newspapers, and in magazines, mailing lists, pamphlet distribution, and word of mouth. Prior to participating in the project, respondents completed an online questionnaire including questions about sociodemographic, socioeconomic data, and health status. The only exclusion criteria were pregnancy, from a foreign country, and not having lived in Italy for at least 20 years. 

The study was conducted in agreement with the Italian ethical requirements on research activities and personal data protection (D.L. 30.6.03 n. 196). The study protocol was approved by the Ethics Committee of Trieste University where the genetics unit of the project was based. The respondents gave their written informed consent at the beginning of the test according to the principles of the Declaration of Helsinki.

### 2.2. Questionnaires

Respondents completed an online questionnaire in order to collect information about their age, gender, country of birth, place of residence, reported height, weight (which were used to calculate the body mass index (BMI) as kg·m^−2^), and their familiarity for a series of food items (*n* = 96) belonging to 13 categories: pasta, vegetables, fruit, legumes, potatoes, extra virgin olive oil, butter, fish, soft drinks, red and cured meat, dairy, sweets, and wine. Food familiarity was assessed using a 5-point labeled scale based on Tuorila et al. [38]: 1 = “I do not recognize it”; 2 = “I recognize it, but I have never tasted it”; 3 = “I have tasted it, but I don’t eat it”; 4 = “I occasionally eat it”; 5 = “I regularly eat it”. This scale covered both the familiarity features of frequency of consumption and levels of product knowledge [30].

Food neophobia was quantified using the Food Neophobia Scale (FNS) developed by Pliner and Hobden [17] and validated in Italian by Laureati et al. [22]. The FNS consists of ten items (five referring to neophilic and five to neophobic statements) evaluated with a 7-point agreement scale ranging from 1 = “I strongly disagree” to 7 = “I strongly agree”. 

### 2.3. Data Analysis

#### 2.3.1. Calculation of the Food Familiarity Index (FFI)

Based on food familiarity and frequency of consumption data, a Food Familiarity Index (FFI) was calculated. The FFI was created by modifying the familiarity/frequency 5-point response [38] in the following three scores, according to Verneau et al. [39]:

- 0: If the response was equal to or less than 3—“I have tasted the product, but I do not consume it”;
- 1: If the response was equal to 4—“I occasionally eat the product”;
- 2: If the response was equal to 5—“I regularly eat the product”.

This modification was done to make the score more discriminating. In fact, since the questionnaire included food items that are generally common in Italy, most of the respondents recognized them as highly familiar (score > 3 on the original 5-point scale).

#### 2.3.2. Calculation of the Italian Taste Mediterranean Index (ITMI)

Since adherence to the MD should be defined according to the specific country’s eating behavior, the Italian Mediterranean Index (IMI), created by Agnoli et al. [1], was chosen as the reference, and adapted to the IT data. Agnoli et al.’s [1] scoring approach is based on the intake of 11 food categories/items: 6 typical Mediterranean foods (pasta, vegetables, fruit, legumes, fish, olive oil); 4 non-Mediterranean foods (butter, soft drinks, red meat, and potatoes); and alcohol. If consumption of typical Mediterranean foods was in the third tertile of the intake distribution, the person received 1 point; otherwise, the person received 0 points. If consumption of non-Mediterranean foods was in the first tertile of the distribution, the person received 1 point; otherwise the person received 0 points. The IMI was then calculated by summing the score received for each food category/item (theoretical range 0–11).

Differently from the IMI questionnaire [1], the IT questionnaire requested participants to indicate the familiarity/frequency use of 96 specific foods belonging to 13 food categories: pasta (6 recipes), vegetables (19 items), fruit (4 items), legumes (4 items), potatoes, extra virgin olive oil (EVOO), butter, fish (8 items), soft drinks (3 items), red and cured meat (16 items), dairy products (17 items), sweets (13 items), and wine (3 items) (for details on the specific items see the Supplementary Materials, Table S1). To generate the ITMI, the same scoring approach of IMI [1] was used for single items (EVOO, butter, potato), while for food categories with multiple items, an average FFI was calculated (e.g., for the pasta category was the average FFI of 6 items, Table S1). For two items that were not considered in the IMI, the following approach was adopted: sweets, only if the participant was in the first tertile of the FFI distribution, did he/she receive 1 point, otherwise 0 points; dairy, when recording moderate use and in the second tertile, the person received 1 point, otherwise 0 points. As related to alcohol, the IMI attributed one point for a consumption of <12 g/day of ethanol (corresponding to one alcoholic unit and a moderate alcohol consumption, according to the Italian Institute of Public Health), while no points were given to both abstainers and persons consuming more than 12 g/day of ethanol. In the present study, wine was chosen as a prototypical Mediterranean alcoholic beverage, according to Trichopoulou et al. [40]. A score of 1 was attributed only to respondents with an FFI within the second tertile. The ITMI resulted from the sum of the adherence scores (theoretical range 0–13). The attribution of adherence scores based on FFI distribution is provided in Table 1.

Reliability of FFI and ITMI was assessed by calculating internal consistency (Cronbach’s α). Analysis of Cronbach’s α with deleted variables was performed in order to investigate whether all the food items/categories contributed in the same way to the construct. Correlations among food items/categories with ITMI were measured using Pearson’s correlation coefficients. 

#### 2.3.3. Calculation of the Food Neophobia Scale (FNS) Score

The individual FNS scores were computed as the sum of the ratings given to the ten statements, after the neophilic items were reversed; thus, the scores theoretically ranged from 10 to 70 with higher scores reflecting higher FN levels. The frequency distribution of FNS scores was calculated and respondents were divided into three groups according to their FN level: low, medium, and high (see details in Section 3.2).

The effect of demographics on food neophobia was explored by 3-way analysis of variance (ANOVA) considering age (18–35, 36–45, 46–60 years), gender, geographical area (NE, NW, CE, SO), and their second-order interactions as fixed factors and food neophobia as the dependent variable. 

The association between ITMI, FFI, FNS score, and background variables (i.e., age, gender, geographical area) was investigated through 4-way ANOVA considering food neophobia level (i.e., low, medium, high), age, gender, geographical area and their second-order interactions as fixed factors and ITMI and FFI for food categories/items as dependent variables. When the ANOVA showed a significant effect (*p* < 0.05), the Bonferroni post-hoc test comparison adjusted for multiple comparison was used.

The SAS/STAT statistical software package version 9.4 (SAS Institute Inc., Cary, NC, USA) and Unscrambler version 11.0 (Camo Analytics AS, Oslo, Norway) were used for the data analysis.

## 3. Results

### 3.1. Characteristics of the Participants

Respondents characteristics are summarized in Table 2. Data were analyzed on 2416 subjects, of which 58.5% (*n* = 1413) were women. The age range was 18–60 years (mean age = 37.7, SD = 13.0). The men’s and women’s mean ages were 37.5 years (SD = 13.3) and 37.8 years (SD = 12.8), respectively. In order to explore possible age-related differences, respondents were divided into three age groups: 18–30 years (*n* = 922, 38.2%); 31–45 years (*n* = 688, 28.5%); and ≥46 years (*n* = 806, 33.3%). They were also grouped according to their geographical origin: 28.6% were from the Northern East; 24.1% from Northern West; 18.2% from the Center; and 29.1% from Southern (including the Islands) of Italy. The proportion of men and women was comparable across age groups (χ^2^ = 1.34, *p* = 0.51) and geographical origin (χ^2^ = 2.20, *p* = 0.53). 

### 3.2. Food Neophobia

Details of the participants according to food neophobia are shown in Table 3. The FNS scores ranged from 10 to 69, covering nearly the possible range (10–70). Overall, the mean FNS score was 27.4 (SEM = 0.24). Based on the FNS score frequency distribution, respondents were divided into three groups according to their food neophobia levels. The low food neophobic group (27.3%) had scores within the lowest quartile ranging from 10 to 18, with a mean value of 14.4. The medium food neophobic group comprised 46.3% of participants, with scores within the second and third quartiles from 19 to 35 and a mean of 26.2. The most neophobic individuals (26.4%) had scores within the highest quartile ranging from 36 to 69 and a mean of 42.9. The ANOVA results showed the significant effects of the main factors: gender (F_1,2398_ = 4.16; *p* = 0.04), age groups (F_2,2398_ = 13.02; *p* < 0.0001), and geographical area (F_3,2398_ = 40.49; *p* < 0.0001) on food neophobia (Table 3). Men had a slightly higher score than women, while food neophobia was significantly higher in the older participants and in the South of Italy. None of the interactions were significant.

### 3.3. Adherence to the MD

Internal consistency of the familiarity/frequency scale calculated by Cronbach’s α was 0.81, much greater than the suggested value of 0.70 given by Nunnally and Bernstein [41]. The correlation between food items/categories and ITMI was positive and highly significant (*p* < 0.0001, except for dairy *p* < 0.01) for pasta, vegetables, fruit, legumes, fish, EVOO, and dairy products; while it was negative and significant for potato, butter, soft drinks, red/cured meat, and sweets with Pearson’s coefficients ranging from 0.10 (dairy) to 0.49 (vegetables) and from −0.20 (potato) to −0.41 (soft drinks) for positive and negative correlations, respectively. Wine was the only category that was not correlated with ITMI. The analysis of Cronbach’s α with deleted variables did not show a significant increase or decrease in the standardized alpha coefficients, suggesting that all food items/categories were strongly correlated with each other and contributed to measure the same construct.

The FFI for each of the 96 food items is reported in Table S1 (Supplementary Materials). The FFI and ITMI by age, gender, geographical area, and food neophobia are reported in Table 4.

Women showed a higher FFI for vegetables, fruit, and legumes, while men consumed significantly more pasta, soft drinks, red/cured meat, and wine (Table 4). This resulted in a significantly higher (F_1,2384_ = 41.08; *p* < 0.0001) ITMI for women (ITMI = 6.70) than for men (ITMI = 6.10). 

The familiarity with/frequency consumption of all typical Mediterranean food items gradually increased with age, while FFI for butter, soft drinks, red/cured meat, and sweets were higher in younger people. Accordingly, ITMI increased systematically and significantly with age (F_2,2384_ = 54.11; *p* < 0.0001). 

Among the Italian regions, SO declared more regular consumption of pasta, vegetables, fruit, legumes, fish, potatoes, soft drink, red/cured meat, and sweets and less dairy products and wine (Table 4), which resulted in a marginal effect (F_3,2384_ = 2.29; *p* = 0.08) of geographical area on ITMI. 

Individuals with high food neophobia showed higher FFIs for soft drinks and sweets and lower FFIs for pasta, vegetables, legumes, fish, extra virgin olive oil, dairy products, and wine than individuals with low food neophobia. As a result, ITMI decreased significantly and systematically with an increase in food neophobia levels (F_2,2384_ = 22.84; *p* < 0.0001). 

There were few significant interactions. Neophobia interacted with age for both vegetables (F_4,2384_ = 4.73; *p* = 0.0008) and legumes (F_4,2384_ = 5.75; *p* < 0.0001) consumption. Vegetables consumption significantly decreased with the food neophobia increase in the two youngest age groups (18–35 and 36–45 years) and in the youngest age group for legumes. 

Food neophobia interacted with geographical area for pasta (F_6,2384_ = 2.38; *p* = 0.03) and fish (F_6,2384_ = 2.12; *p* = 0.05), in particular, the familiarity/consumption of pasta and fish decreased with the increase of neophobia only in the Northern regions. The familiarity/consumption of fish was also influenced by the geographical area×age interaction (F_6,2384_ = 2.13; *p* = 0.05). In general, fish was more regularly consumed in both the Center and South of Italy than in the Northern regions, and in the Southern region, its consumption increased with age. 

The familiarity/consumption of soft drinks was influenced by the gender×geographical area interaction (F_3,2384_ = 3.72; *p* = 0.01) with women from Southern Italy and men from the Center and South of Italy having the highest scores.

Wine familiarity/consumption was influenced by gender×age interaction (F_2,2384_ = 7.00; *p* = 0.0009). Scores increased with age in both genders, but the increase was steeper in men than women.

Finally, the only significant interaction influencing ITMI was age×neophobia (F_4,2384_ = 3.27; *p* = 0.01). In the two youngest groups, ITMI decreased significantly with the increase of food neophobia, while ITMI in the oldest group was comparable within neophobia level and was as high as for the neophilic subjects of the other two age groups. 

## 4. Discussion

Several studies have investigated the Mediterranean eating pattern and its association with human health, but relatively little information exists about its interplay with personality traits. The present study explored the relation between the MD, food neophobia, and sociodemographic factors, including age, gender and geographical origin, which are known to play an important role in food selection and diet quality, considering a large population sample of Italian consumers.

The present study made an innovative contribution to the scientific literature in at least two ways. First, we calculated a new index of adherence to the MD, recording familiarity/frequency scores for specific food items and popular gastronomic preparations, not just food categories, permitting a wider and more detailed approach to food choices analyzed within a given food culture framework. This new index was found to be an adequate tool for investigating the MD and identifying some of the main determinants of its adherence, namely, gender and age. In this respect, we found a higher adherence to the MD in women and in older people. Second, the present study was the first to provide evidence of a strong, negative association between adherence to the MD and food neophobia in a large population sample of adults, being previous studies mainly focused on children and adolescents [32,33].

The Italian population participating in this study showed familiarity and more regular use of food categories, providing data consistent with previous research. Specifically, the very frequent use of EVOO, as well as the regular consumption of fruit, vegetables, legumes and pasta were in agreement with studies at regional level [42,43].

Moreover, women recorded a more regular use of fruit, vegetables and legumes, and a less frequent use of soft drinks, meat, pasta, and wine than men. These findings are in accordance with previous studies in Italy, indicating that animal protein intake is higher in men [44] than in women, who also consume fruit and vegetables more regularly [45]. A low adherence to the MD in men has been previously reported and correlated with markers of atherosclerosis and cardiovascular risk [46]. In addition, the present study showed that men have a more regular use of cured meat, generally considered as a food with unbalanced nutritional value due to the high fat and salt content [47]. Studies in the elderly showed that older men tend to have poorer dietary intakes including consumption of fewer fruits and vegetables than women of the same age [48,49]. Moreover, in our study, we found that young people (18–30 years) had the lowest familiarity with consumption of pasta, fruit, vegetables, legumes, EVOO, and fish and a lower adherence to the MD. Age- and gender-related differences in the ITMI are in line with previous results indicating that women have higher interest in health and natural products and that people are more committed about consuming healthy foods as they age [50,51,52]. This would suggest that dietary strategies should be planned according to gender- and age-related food habits.

Only slight differences emerged among regional areas, with the South and Islands prevailing for regular use of typical Mediterranean food, such as fish, and together with the Center, pasta, vegetables, fruits, and legumes, but this was counterbalanced by relevant use of potatoes and red/cured meat. Congruent data about South Italy’s food habits were reported in a study comparing different Italian areas [53].

Data indicated a strong negative association between food neophobia and adherence to the MD. While this result was in agreement with the well-known negative association of food neophobia with the liking and consumption of vegetables, fruit, fish, and wine [21,22,54], it suggested that people with a higher level of food neophobia may adopt less healthy dietary patterns and have a greater metabolic disease risk. In line with our findings, Maiz and Balluerka [32] reported that neophobic children and adolescents presented a poorer quality of the Mediterranean diet due to the lower intake of fruit, vegetables, and fish and a higher intake of sweets or candy.

Interestingly, we found that, with increasing age, ITMI increases even in individuals with a high level of food neophobia. This result can be explained by greater attention, increasing with age, to factors related, for instance, to the consumption of healthy foods that may counterbalance the effect of neophobia on the adherence to the MD [52].

Data found in this Italian cohort confirm the association between food neophobia and demographic factors, including age, gender, and living area, as previously found in large-scale studies. Considering the effect of gender on food neophobia, contradicting results are found in the literature. The analysis of nationally representative studies involving adults either showed no effect [54] or a weak effect of gender on food neophobia with men being more neophobic than women [38,55,56].

Results on the effect of age are more consistent and highlight higher neophobia levels with increasing age [26,38,56]. Living area is another variable which might affect exposure to new and unusual products. Meiselman et al. [26] and Tuorila et al. [38] reported that food neophobia declines with urbanization, since the inhabitants of a rural area may have fewer opportunities to be exposed to new and unusual foods (e.g., ethnic foods). This may probably explain the result of a higher food neophobia level in the Southern regions of Italy found in the present study.

Finally, our data confirm that individuals with higher levels of food neophobia have lower familiarity and frequency indices for ingredients and foods which are very popular within the Italian population and typical of the MD. Jaeger et al. [21], in a large population study in New Zealand, also reported that food neophobia decreased the consumption of common place food; they explained the outcome by the fact that individuals with high food neophobia levels may include not only those who have a fear of new foods but also those having little interest in foods [38] and/or less positive associations with food throughout their lives.

The large size and geographical diversity of our sample, balanced for age and gender, make us confident about the external validity of the results. Biased sources in evaluating food consumption behavior may derive from food choices conditioned by health, ethical or religious factors. One limitation of the study was that we did not control for potential bias deriving from medications and comorbidities declared by the respondents. On the other hand, the participants were recruited randomly, and the population was not subjected to selections to preserve the representativeness of the sample and of related data. Another limitation of this study was the bias resulting from the self-reported data (misunderstandings, memory bias, etc.). The ITMI was obtained by self-reported consumption habits of food items (non-use, occasional use, regular use) and, therefore, not from a record of real consumption over a temporal range. Weekly dietary records are believed to be a more accurate measure, although data from diaries are also not error-free, including infrequency and fatigue.

## 5. Conclusions

In this study, a new adherence index to the MD was developed in order to explore, in a large Italian population cohort, the relationships between dietary pattern and food neophobia as affected by sociodemographic characteristics. 

Data indicated that food neophobia was inversely associated with adherence to the MD, suggesting that this personality trait may affect dietary pattern, lowering its potential health benefits. In our population, this behavior might be relevant mainly in men, elderly people, and individuals from Southern Italy, as they showed higher levels of food neophobia. 

Moreover, our results suggest that the concept and the meaning of food neophobia should be reconsidered, because it is not a phenomenon limited to the pediatric population and to the rejection of new and unfamiliar foods, but, rather, it is more generalized, also embracing common and familiar foods and extending beyond childhood. Therefore, the effects of this behavioral trait on individual diet and health could have potentially been underestimated so far and needs further attention. Population interventions, preferably starting at an early age, aimed at reducing food neophobia, combining dietary education and strategies to manage anxiety, which has been shown to be a predictor of neophobia [17,57], may be a valuable strategy to control the negative effect of this personality trait on individuals and its effect on public nutrition. 

## Figures and Tables

**Table 1:** Attribution of adherence scores based on the tertile distribution of the individual Food Familiarity Index (FFI). (EVOO = extra virgin olive oil).

Food Item/Category	FFI in 1st Tertile	FFI in 2nd Tertile	FFI in 3rd Tertile
Pasta	0	0	1
Vegetables	0	0	1
Fruit	0	0	1
Legumes	0	0	1
Fish	0	0	1
EVOO	0	0	1
Potato	1	0	0
Butter	1	0	0
Soft drinks	1	0	0
Red and cured meat	1	0	0
Sweets	1	0	0
Dairy	0	1	0
Wine	0	1	0

**Table 2:** Characteristics of the participants.

Variables	Men (*n* = 1003) %	Women (*n* = 1413) %	Total (*n* = 2416) %
**Gender**	41.5	58.5	100
**Age (years)**			
18–30	39.5	37.2	38.2
31–45	27.6	29.1	28.5
≥46	32.9	33.7	33.3
**Geographical areas (Italy)**			
North East	30.0	27.5	28.6
North West	24.0	24.1	24.1
Center	17.3	18.8	18.2
South and Islands	28.7	29.6	29.1

**Table 3:** Effect of gender, age, and geographical area (NW = North West, NE = North East; CE = Center; SO = South and Islands) on FNS (Food Neophobia Scale) score and details of the participants grouped according to food neophobia level (low, medium, and high).

Variable	Categorization	FNS Score		SEM	*p*-Value
		Range	Mean		
Gender	Women	10–69	26.7 ^b^	0.30	0.04
	Men	10–62	27.7 ^a^	0.36	
Age (years)	18–30	10–69	26.3 ^b^	0.38	<0.0001
	31–45	10–58	26.4 ^b^	0.45	
	46–60	10–64	28.9 ^a^	0.40	
Geographical area	NW	10–65	25.5 ^b^	0.48	<0.0001
	NE	10–60	25.7 ^b^	0.44	
	CE	10–66	26.3 ^b^	0.44	
	SO	10–69	31.4 ^a^	0.43	
FNS	Low	10–18	14.4	0.10	
	Medium	19–35	26.2	0.15	
	High	36–69	42.9	0.44	
	All	10–69	27.4	0.24	

Different letters indicate significant differences according to Bonferroni post-hoc test.

**Table 4:** Total Food Frequency Index (FFI, range 0–2) and FFI calculated for each food category by gender (F: females; M: males), age group, geographical area, food neophobia level, and derived Italian Taste Mediterranean Index (ITMI, range 0–13). Statistical differences were determined for gender, age groups, geographical area (NE = North-East; NW = North-West; CE = Centre; SO = South and Islands), and food neophobia level (i.e., low, medium, high).

Food Category		Gender			Age Groups (years)				Geographical Area					Food Neophobia			
	Total FFI	F	M	*p*	18–30	31–45	≥46	*p*	NE	NW	CE	SO	*p*	Low	Medium	High	*p*
Pasta	1.05	1.00 ^b^	1.09 ^a^	***	1.02 ^b^	1.04 ^ab^	1.09 ^a^	**	0.95 ^b^	0.97 ^b^	1.12 ^a^	1.16 ^a^	***	1.09 ^a^	1.07 ^a^	1.00 ^b^	***
Vegetables	1.31	1.37 ^a^	1.24 ^b^	***	1.17 ^c^	1.32 ^b^	1.42 ^a^	***	1.28 ^b^	1.27 ^b^	1.32 ^ab^	1.34 ^a^	**	1.38 ^a^	1.32 ^b^	1.22 ^c^	***
Fruit	1.33	1.36 ^a^	1.30 ^b^	**	1.28 ^b^	1.34 ^ab^	1.38 ^a^	***	1.29 ^b^	1.32 ^ab^	1.33 ^ab^	1.39 ^a^	**	1.33	1.35	1.32	
Legumes	1.42	1.47 ^a^	1.37 ^b^	***	1.31 ^c^	1.44 ^b^	1.52 ^a^	***	1.36 ^c^	1.37 ^bc^	1.43 ^ab^	1.53 ^a^	***	1.46 ^a^	1.45 ^a^	1.36 ^b^	***
Fish	0.87	0.88	0.86		0.82 ^b^	0.88 ^a^	0.91 ^a^	***	0.79 ^c^	0.83 ^bc^	0.88 ^b^	0.97 ^a^	***	0.93 ^a^	0.88 ^b^	0.80 ^c^	***
EVOO	1.90	1.90	1.89		1.86 ^b^	1.90 ^b^	1.93 ^a^	***	1.88	1.90	1.92	1.90		1.92	1.90	1.87	
Potato	1.40	1.40	1.39		1.37	1.42	1.40		1.35 ^bc^	1.31 ^c^	1.42 ^b^	1.50 ^a^	***	1.40	1.40	1.39	
Butter	1.09	1.06	1.11		1.11 ^a^	1.11 ^a^	1.03 ^b^	*	1.10	1.04	1.11	1.09		1.12	1.08	1.00	
Soft Drinks	0.77	0.72 ^b^	0.82 ^a^	***	0.88 ^a^	0.74 ^b^	0.64 ^b^	***	0.71 ^b^	0.72 ^b^	0.81 ^b^	0.85 ^a^	***	0.72 ^b^	0.74 ^b^	0.85 ^a^	***
Red/cured meat	1.02	0.98 ^b^	1.06 ^a^	***	1.04 ^a^	1.05 ^a^	0.97 ^b^	**	0.95 ^c^	0.97 ^c^	1.04 ^b^	1.12 ^a^	***	1.01	1.01	1.04	
Sweets	0.85	0.84	0.85		0.86 ^a^	0.86 ^a^	0.81 ^b^	**	0.82 ^b^	0.83 ^b^	0.88 ^a^	0.86 ^a^	*	0.82 ^b^	0.84 ^ab^	0.88 ^a^	*
Dairy	0.91	0.92	0.90		0.87 ^b^	0.91 ^a^	0.94 ^a^	***	0.85 ^b^	0.87 ^b^	0.97 ^a^	0.93 b	***	0.95 ^a^	0.91 ^ab^	0.86 ^b^	***
Wine	0.88	0.77 ^b^	1.00 ^a^	***	0.81 ^c^	0.89 ^b^	0.98 ^a^	***	0.91 ^ab^	0.97 ^a^	0.88 ^ab^	0.82 ^b^	***	0.99 ^a^	0.91 ^b^	0.78 ^c^	***
ITMI	6.55	6.70 ^a^	6.10 ^b^	***	5.97 ^c^	6.42 ^b^	6.95 ^a^	***	6.30	6.48	6.42	6.58		6.76 ^a^	6.53 ^b^	6.03 ^c^	***

Different letters by row correspond to significantly different means according to the Bonferroni post-hoc test (* *p* < 0.05; ** *p* < 0.01; *** *p* < 0.001).

## Supplementary Materials

The following are available online at https://www.mdpi.com/2072-6643/12/6/1778/s1, Table S1: Food Familiarity index (FFI) calculated by food item.
