# Supplementary material for: Gender, Age, Geographical Area, Food Neophobia and Their Relationships with the Adherence to the Mediterranean Diet: New Insights from a Large Population Cross-Sectional Study
Source: Nutrients. 2020 Jun 15;12(6):1778. doi: 10.3390/nu12061778 (PMC7353239; doi:10.3390/nu12061778)
Supplement: Supplementary file 1 [file nutrients-12-01778-s001.pdf]

Table S1. Food Familiarity index (FFI, range 0-2) calculated by food item. Statistical differences are determined according to 2-way ANOVA for gender, age groups, geographical area (NE= North-East, NW= North-West, CE= Center, SO= South and Islands), and food neophobia (p<0.05; ** p<0.01; *** p<0.0001)*

**Food items**		**Gender**			**Ager groups (years)**				**Geographical area**					**Food neophobia**			
**Pasta**	**All**	**W**	**M**	**p-value**	**18-30**	**31-45**	**≥ 46**	**p-value**	**NE**	**NW**	**CE**	**SO**	**p-value**	**Low**	**Med**	**High**	**p-value**
Spaghetti tomato	1,53	1,51	1,56	*	1,52	1,51	1,56		1,45	1,49	1,50	1,66	***	1,48	1,53	1,58	
Spagh. Spicy tomato	0,83	0,76	0,93	***	0,79	0,83	0,86		0,79	0,81	0,95	0,81	***	0,91	0,84	0,73	***
Spagh. garlic, oil, hot pepper	1,02	0,95	1,11	***	0,95	1,04	1,08	**	0,93	0,92	1,11	1,12	***	1,05	1,02	0,99	**
Spaghetti garlic, oil	1,00	0,96	1,06	***	0,94	0,99	1,09	**	0,85	0,88	1,09	1,20	***	1,00	0,99	1,04	
Spaghetti seafood	0,98	0,98	0,98		0,95	0,98	1,02		0,90	0,89	1,05	1,09	***	0,99	0,96	0,99	
Pasta zucchini-shrimps	0,94	0,97	0,89		0,92	0,96	0,94		0,84	0,90	1,00	1,03	***	0,98	0,95	0,88	***
**Vegetables**																	
Artichoke	1,28	1,34	1,18	***	1,08	1,32	1,46	***	1,19	1,21	1,35	1,37	***	1,33	1,26	1,25	***
Asparagus	1,14	1,17	1,10		0,96	1,21	1,29	***	1,19	1,22	1,17	1,01	***	1,25	1,15	1,02	***
Beet	1,07	1,17	0,94	***	0,82	1,14	1,30	***	1,03	0,85	1,27	1,16	***	1,15	1,06	1,02	***
Broccoli	1,34	1,43	1,22	***	1,17	1,39	1,50	***	1,24	1,37	1,36	1,41	***	1,47	1,35	1,21	***
Carrot	1,37	1,46	1,25	***	1,30	1,40	1,43	***	1,40	1,39	1,27	1,39	**	1,42	1,37	1,32	
Cauliflower	1,03	1,12	0,89	***	0,85	1,05	1,21	***	1,01	1,04	0,96	1,07	**	1,13	1,01	0,95	***
Courgettes	1,68	1,76	1,58	***	1,61	1,71	1,75	***	1,66	1,71	1,69	1,67		1,74	1,69	1,61	***
Cucumber	1,00	1,06	0,93	***	0,92	1,04	1,07	**	1,13	0,81	1,10	0,98	***	1,11	0,99	0,92	***
Chicory	0,88	0,91	0,83		0,66	0,93	1,08	***	0,90	0,82	0,99	0,83		0,96	0,86	0,82	**
Eggplants grilled	1,34	1,42	1,23	***	1,24	1,40	1,41	***	1,24	1,29	1,36	1,48	***	1,43	1,32	1,31	***
Eggplants Parmesan	1,06	1,06	1,07		0,98	1,10	1,14	***	0,93	0,96	1,03	1,31	***	1,06	1,05	1,09	
Fennel	1,41	1,51	1,27	***	1,24	1,47	1,56	***	1,34	1,46	1,41	1,44	*	1,46	1,40	1,38	*
Garlic	1,38	1,38	1,37		1,29	1,40	1,46	***	1,27	1,27	1,50	1,49	***	1,44	1,38	1,31	***
Lattuce	1,45	1,54	1,32	***	1,37	1,43	1,56	***	1,52	1,55	1,51	1,25	**	1,54	1,45	1,35	*
Onion	1,58	1,60	1,57		1,53	1,61	1,62	*	1,52	1,59	1,62	1,63	**	1,67	1,60	1,48	***
Radicchio	1,14	1,18	1,09	*	0,96	1,16	1,34	***	1,26	1,07	1,23	1,04	***	1,21	1,15	1,06	*
Spinach	1,47	1,54	1,37	***	1,39	1,48	1,55	***	1,42	1,53	1,51	1,45	*	1,53	1,49	1,38	***
Tomato	1,75	1,78	1,72	*	1,70	1,75	1,82	***	1,72	1,74	1,78	1,78		1,77	1,75	1,75	
Vegetable soup	1,52	1,61	1,39	***	1,40	1,53	1,64	***	1,50	1,55	1,50	1,51		1,55	1,53	1,46	*

**Fruit**																	
Tangerine	1,53	1,57	1,46	***	1,46	1,55	1,59	***	1,44	1,49	1,57	1,63	***	1,53	1,52	1,54	
Pear	1,37	1,40	1,34		1,29	1,38	1,47	***	1,33	1,39	1,36	1,42		1,38	1,37	1,39	
Yellow apple	1,38	1,45	1,32	***	1,33	1,38	1,48	***	1,38	1,41	1,35	1,42		1,37	1,40	1,41	
Green apple	1,04	1,04	1,05		1,08	1,06	0,99	*	1,01	1,02	1,08	1,08		1,04	1,06	1,02	
**Legumes**																	
Beans	1,42	1,41	1,42		1,29	1,46	1,52	***	1,35	1,29	1,45	1,57	***	1,42	1,43	1,40	**
Peas	1,48	1,53	1,41	***	1,42	1,53	1,52	***	1,42	1,47	1,47	1,56	**	1,49	1,49	1,47	
Green bean	1,46	1,52	1,36	***	1,31	1,48	1,60	***	1,38	1,46	1,51	1,49	*	1,51	1,45	1,41	**
Legumes soup	1,37	1,43	1,29	***	1,26	1,41	1,48	***	1,30	1,34	1,38	1,47	***	1,43	1,37	1,33	**
**Fish**																	
Anchovy	0,74	0,73	0,76		0,60	0,79	0,86	***	0,62	0,66	0,71	0,93	***	0,76	0,72	0,75	**
Cod	1,06	1,12	0,99	***	1,00	1,09	1,12	***	0,96	0,96	1,09	1,23	***	1,08	1,06	1,04	*
Salted codfish	0,71	0,71	0,71		0,54	0,80	0,83	***	0,63	0,64	0,78	0,82	***	0,76	0,68	0,73	**
Shrimps	0,69	0,70	0,67		0,66	0,71	0,70		0,63	0,67	0,71	0,65	**	0,74	0,67	0,66	**
Prawns	0,97	0,99	0,95		0,94	1,01	0,97		0,90	0,93	0,97	1,07	***	1,03	0,96	0,92	***
Sole	0,88	0,92	0,83	**	0,80	0,92	0,94	***	0,84	0,87	0,88	0,94	*	0,92	0,87	0,88	
Salmon	1,01	1,03	0,99		1,04	1,01	0,98		0,97	1,03	1,06	1,01	*	1,09	1,04	0,88	***
Tuna	0,93	0,94	0,93		0,99	0,92	0,89	**	0,87	0,94	0,94	0,99	***	1,00	0,93	0,88	***
**Butter**	1,08	1,06	1,11		1,11	1,11	1,03	*	1,10	1,04	1,11	1,09		1,12	1,08	1,00	
**EVOO**	1,90	1,90	1,89		1,86	1,89	1,93	***	1,88	1,90	1,92	1,90		1,92	1,90	1,87	
**Potato**	1,40	1,40	1,39		1,37	1,42	1,40		1,35	1,31	1,42	1,50	***	1,40	1,40	1,39	
**Soft Drinks**																	
Carbonated Drinks	0,79	0,72	0,89	***	0,86	0,80	0,70	***	0,72	0,76	0,79	0,88	***	0,73	0,76	0,90	***
Lemon Ice Tea	0,80	0,77	0,84	**	0,88	0,75	0,74	***	0,74	0,71	0,87	0,89	***	0,73	0,78	0,90	**
Peach Ice Tea	0,69	0,64	0,75	***	0,84	0,60	0,58	***	0,65	0,62	0,64	0,80	***	0,63	0,67	0,78	**
**Meat**																	
Beef Steak	1,24	1,20	1,28	**	1,23	1,25	1,22		1,19	1,21	1,22	1,31	*	1,21	1,21	1,31	*
Beef ribs	0,93	0,90	0,97	**	0,90	0,73	0,89	*	0,74	0,86	0,92	1,19	***	0,86	0,92	1,03	
Bresaola	1,14	1,17	1,10	*	1,10	1,18	1,15	n.s.	1,04	1,16	1,16	1,21	***	1,13	1,16	1,12	
Cutlet	1,01	0,97	1,07	**	1,06	1,02	0,95	***	0,91	0,95	0,96	1,20	***	0,93	1,01	1,11	***
Hamburger	0,81	0,77	0,86	***	0,99	0,84	0,57	***	0,66	0,71	0,87	0,90	***	0,85	0,78	0,81	
Bacon	0,92	0,86	1,01	***	0,96	0,92	0,89		0,89	0,85	0,93	1,02	***	0,92	0,91	0,95	
Pork chop	1,03	0,97	1,10	***	1,03	1,04	1,01		1,00	0,89	1,17	1,07	***	1,01	1,00	1,09	*

Lamb Ribs	0,67	0,62	0,75	***	0,63	0,73	0,67	*	0,55	0,56	0,64	0,90	***	0,7	0,64	0,70	*
Pork Sausage	1,11	1,04	1,22	***	1,11	1,16	1,07		1,01	0,96	1,12	1,34	***	1,08	1,10	1,18	
Carpaccio	0,72	0,68	0,77	***	0,67	0,79	0,72	**	0,74	0,33	0,73	0,60	***	0,77	0,72	0,67	
Veal steak	1,17	1,17	1,17		1,18	1,27	1,06	***	0,98	1,12	1,19	1,37	***	1,12	1,14	1,26	*
Bologna	0,97	0,92	1,03	***	0,96	1,00	0,94		0,91	0,82	1,03	1,11	***	0,93	0,94	1,06	*
Cured Ham	1,46	1,47	1,45		1,45	1,46	1,47		1,44	1,36	1,49	1,54	***	1,41	1,47	1,49	
Cooked Ham	1,32	1,35	1,28	*	1,36	1,34	1,26	*	1,27	1,34	1,25	1,40	*	1,25	1,33	1,39	**
Salami	1,07	0,99	1,18	***	1,10	1,10	1,02	*	1,01	1,04	1,05	1,17	**	1,05	1,04	1,15	*
Spicy sausage	0,63	0,54	0,75	***	0,63	0,63	0,62		0,59	0,55	0,69	0,69	***	0,68	0,60	0,63	**
**Dairy**																	
Gorgonzola	0,84	0,82	0,86		0,73	0,86	0,94	***	0,87	1,01	0,87	0,65	***	0,98	0,83	0,70	***
Milk flacks	0,44	0,50	0,34	***	0,44	0,48	0,39	*	0,41	0,37	0,51	0,47	**	0,50	0,40	0,43	
Mozzarella (buffalo)	1,14	1,14	1,14		1,10	1,16	1,18		1,05	1,09	1,20	1,24	***	1,13	1,15	1,14	
Mozzarella (cow)	1,31	1,35	1,27	**	1,25	1,32	1,37	*	1,27	1,29	1,35	1,35		1,31	1,31	1,33	
Parmigiano	1,61	1,62	1,59		1,52	1,61	1,70	***	1,56	1,63	1,63	1,62		1,61	1,62	1,59	
Pecorino cheese	0,88	0,86	0,92	*	0,79	0,92	0,96	***	0,80	0,77	1,17	0,88	***	0,98	0,88	0,78	***
Ricotta cheese	1,10	1,18	0,97	***	1,00	1,11	1,19	***	1,02	1,04	1,16	1,17	***	1,15	1,06	1,11	
Skimmed Milk	0,93	0,97	0,88	*	1,00	0,86	0,92	*	0,89	0,88	0,93	1,02		0,86	0,95	0,98	*
Spicy Provolone	0,59	0,54	0,66	***	0,51	0,65	0,63	***	0,43	0,48	0,67	0,78	***	0,63	0,56	0,61	*
Stracchino cheese	1,04	1,09	0,98	***	0,97	1,04	1,13	**	1,10	1,09	1,28	0,80	***	1,14	1,03	0,96	*
Sweet Provolone	0,77	0,76	0,78		0,72	0,81	0,79		0,56	0,65	0,80	1,04	***	0,77	0,74	0,82	
Whole milk	0,80	0,73	0,90	***	0,79	0,80	0,82		0,86	0,80	0,85	0,73	*	0,80	0,84	0,74	
Yogurt agrumes	0,62	0,62	0,63		0,56	0,68	0,65	**	0,59	0,61	0,66	0,65		0,61	0,62	0,65	
Yogurt light	0,85	0,93	0,74	***	0,83	0,84	0,87		0,83	0,79	0,91	0,87	*	0,92	0,84	0,78	**
Yogurt vanilla	0,60	0,61	0,60		0,64	0,62	0,55	**	0,63	0,55	0,66	0,59		0,60	0,61	0,60	
Yogurt whole	0,88	0,94	0,80	***	0,85	0,88	0,92		0,93	0,86	0,92	0,83		0,90	0,88	0,77	***
Aged Pecorino cheese	0,94	0,89	1,01	***	0,84	0,96	1,04	***	0,87	0,88	1,16	0,93	***	1,06	0,93	0,83	***
**Wine**																	
Red wine	1,05	0,91	1,25	***	0,93	1,07	1,17	***	1,03	1,14	1,03	1,00	*	1,14	1,06	0,93	***
White Wine	0,81	0,72	0,93	***	0,75	0,81	0,88	***	0,88	0,89	0,84	0,65	***	0,93	0,82	0,66	***
Sparkling Wine	0,76	0,70	0,85	***	0,72	0,73	0,83	***	0,83	0,82	0,73	0,67	***	0,81	0,77	0,69	**
**Sweets**																	
Apple pie	0,64	0,61	0,67	**	0,62	0,62	0,68		0,62	0,60	0,67	0,67		0,61	0,63	0,68	
Biscuits	1,26	1,30	1,20	***	1,26	1,23	1,27		1,23	1,24	1,29	1,28		1,22	1,27	1,27	
Butter cookies	0,80	0,84	0,74	**	0,82	0,83	0,74	*	0,77	0,74	0,86	0,83	*	0,80	0,80	0,79	

Chocolate Icecream	0,90	0,88	0,92		0,86	0,93	0,91		0,85	0,84	0,96	0,94	*	0,87	0,89	0,93	
Chocolate pudding	0,52	0,50	0,54	*	0,50	0,53	0,51		0,52	0,59	0,54	0,44	***	0,53	0,49	0,54	*
Croissant	0,76	0,80	0,70	**	0,76	0,80	0,73		0,65	0,66	0,93	0,84	***	0,72	0,72	0,87	**
Croissant stuffed	0,92	0,89	0,96	*	1,01	0,95	0,78	***	0,89	0,84	0,90	1,02	***	0,92	0,89	0,96	
Milk Chocolate	0,93	0,92	0,93		1,02	0,92	0,83	***	0,90	0,85	0,87	1,05	**	0,87	0,90	1,03	***
Dark Chocholate	1,14	1,16	1,12		1,07	1,18	1,19	***	1,14	1,23	1,16	1,07	*	1,20	1,15	1,06	**
Fruit pie	0,95	0,95	0,96		0,91	0,98	0,98	*	0,93	0,96	0,97	0,95		0,92	0,96	0,97	
Panna cotta	0,59	0,57	0,61		0,57	0,62	0,58		0,61	0,61	0,65	0,52	***	0,62	0,59	0,57	
Sweet snacks	0,67	0,65	0,70		0,80	0,70	0,49	***	0,59	0,62	0,62	0,84	***	0,61	0,64	0,79	***
Tiramisù	0,90	0,90	0,93		0,96	0,90	0,84	***	0,85	0,87	0,91	0,98	***	0,87	0,91	0,93	
